# Dental follicle progenitor cells responses to *Porphyromonas gingivalis* LPS

**DOI:** 10.1111/jcmm.12058

**Published:** 2013-04-08

**Authors:** Kyriaki Chatzivasileiou, Cornelia A Lux, Gustav Steinhoff, Hermann Lang

**Affiliations:** a Department of Conservative Dentistry and Periodontology, University of RostockRostock, Germany; b Reference and Translation Center for Cardiac Stem Cell Therapy (RTC), University of RostockRostock, Germany

**Keywords:** stem cells, dental follicle, *Porphyromonas gingivalis*, lipopolysaccharide, toll-like receptors, wound healing, interleukin-6

## Abstract

Periodontitis is a bacterially induced chronic inflammatory disease. Dental follicle progenitor cells (DFPCs) have been proposed as biological graft for periodontal regenerative therapies. The potential impact of bacterial toxins on DFPCs properties is still poorly understood. The aim of this study was to investigate whether DFPCs are able to sense and respond to lipopolysaccharide (LPS) from *Porphyromonas gingivalis*, a major periopathogenic bacterium. Specifically, we hypothesized that LPS could influence the migratory capacity and IL-6 secretion of DFPCs. DFPCs properties were compared to bone marrow mesenchymal stem cells (BMSCs), a well-studied class of adult stem cells. The analysis by flow cytometry indicated that DFPCs, similar to BMSCs, expressed low levels of both toll-like receptor (TLR) 2 and 4. The TLR4 mRNA expression was down-regulated in response to LPS in both cell populations, while on protein level TLR4 was significantly up-regulated on BMSCs. The TLR2 expression was not influenced by the LPS treatment in both DFPCs and BMSCs. The migratory efficacy of LPS-treated DFPCs was evaluated by *in vitro* scratch wound assays and found to be significantly increased. Furthermore, we assayed the secretion of interleukin-6 (IL-6), a potent stimulator of cell migration. Interestingly, the levels of IL-6 secretion of DFPCs and BMSCs remained unchanged after the LPS treatment. Taken together, these results suggest that DFPCs are able to sense and respond to *P. gingivalis* LPS. Our study provides new insights into understanding the physiological role of dental-derived progenitor cells in sites of periodontal infection.

## Introduction

In recent years it has been shown that periodontitis is an inflammatory disease mainly caused by the presence of an oral microbial biofilm. An inadequate host inflammatory-immune response to periodontal pathogens leads ultimately to progressive destruction of the periodontium in the pathogenesis of the disease 1. The ultimate goal of periodontal treatment is to arrest the disease process and promote the regeneration of lost periodontal supporting tissues 2. Although the available periodontal therapies may result in improved clinical outcomes, it remains insufficient to achieve complete and predictable periodontal regeneration 3. Currently, emphasis has been given on exploring the biological processes involved in the formation and regeneration of the periodontium. Cell-based approaches based on transplantation of bone marrow-derived stromal cells and periodontal ligament stem cells have been proposed as promising alternatives to conventional treatments 4. Unfortunately, these new regenerative techniques are clinically unreliable, resulting in only partial regeneration at best 5. Recently, multipotent cells derived from dental tissues have been proposed as suitable source for such cellular therapies 6. Particularly, dental follicle progenitor cells (DFPCs) are thought to contribute to the formation of all periodontal tissues, namely cementum, periodontal ligament and alveolar bone 7. Such cells could therefore play a key role in achieving the promise of periodontal regeneration. In 2005, Morsczeck *et al*. were able to isolate multipotent cells from the dental follicle of human impacted third molars and describe their stem cell characteristics 8.

The growing interest in using progenitor cells for therapies against infectious diseases like periodontitis implies that the potential impact of bacterial toxins on cell properties warrants further research 9. Recent reports documented the ability of dental-derived progenitor cells to recognize pathogen-associated molecular patterns (PAMPs) 10, 11. However, the modulation of progenitor cell properties after exposure to PAMPs remains poorly understood. *Porphyromonas gingivalis* LPS is a crucial virulence factor strongly involved in the initiation and development of periodontal disease 12. Particularly, it has been reported that *P. gingivalis* LPS acts as a potent stimulator of inflammatory cytokine production and bone resorption 13. Two members of the toll-like receptor family, TLR2 and TLR4, have been identified as possible signalling receptors for *P. gingivalis* LPS 14. Until now little is known about the ability of DFPCs to express TLRs for LPS sensing. Furthermore, the immunomodulation along with the migratory ability of stem cells are considered to play an important role in their therapeutic efficacy 15. Thus, a better understanding of the effects of toxins on DFPCs basal motility and cytokine secretion profile could be critical for their successful application.

In this study, we hypothesize that human DFPCs are able to sense and respond to *P. gingivalis* LPS. We sought to comparatively investigate the effects of *P. gingivalis* LPS on TLRs expression, migratory efficiency, cell viability and cytokine secretion of DFPCs and bone marrow mesenchymal stem cells (BMSCs).

## Materials and methods

### Isolation and culture of human DFPCs and BMSCs

Healthy human impacted third molars (*n* = 6) were surgically removed and collected from patients (aged 17–23 years) at the Dental School of the University of Rostock, following approved guidelines set by the commission of ethics of the Medical School of Rostock (Reg. Nr: A 2010 87). The freshly extracted dental follicles were separated from the mineralized tooth. Followingly, dental follicle tissues were minced and digested in a solution of 0.1 U/ml Collagenase and 0.8 U/ml Dispase (Roche, Mannheim, Germany) for 1 hr at 37°C. Explants were then transferred to T25 cell culture flasks and cultivated in MSCGM medium (Lonza, Walkersville, MD, USA) at 37°C in 5% CO_2_ humidified atmosphere. Single cells had attached to the plastic surface within 24 hrs, after which non-adherent cells were removed and culture medium was replaced every 2–3 days. Cells from passages 1 to 3 were used for all experiments.

Human mesenchymal stem cells processed from bone marrow aspirates of human adult volunteers (*n* = 8) were isolated and prepared as previously described 16. Informed consent was provided according to the Declaration of Helsinki. Cells were washed and cultivated in MSCGM. BMSCs from passages 1 to 3 were used for the subsequent *in vitro* experiments.

### Colony-forming assay

Human DFPCs and BMSCs at passage 1 were cultured to confluence and detached by 0.05% (w/v) trypsin and 0.02% (w/v) EDTA. Single-cell suspensions were then seeded at low densities (30 cells per cm^2^) into 6-well plates. After 12 days of incubation, cells were fixed with 4% PFA and washed with distilled water. The total number of colonies was determined microscopically (Axiovert 40 CFL; Carl Zeiss, Goettingen, Germany), by scoring aggregates of more than 50 cells. The percentage of colony-forming efficiency (CFE) was calculated as follows: CFE (%) = (no of colonies formed/no of cells seeded) × 100%.

### 3-2,5-Diphenyltetrazolium bromide (MTT) dye reduction assay

To determine the metabolic activity of cells, MTT assays were performed. Cells were seeded in 96-well plates at a density of 1 × 10³ cells per well in MSCGM. Wells containing culture medium only served as blank controls for non-specific dye reduction. For the measurement MTT solution was added to each well to a final concentration of 0.5 mg/ml. After 4 hrs of incubation at 37°C, the medium was removed and the formazan crystals dissolved in DMSO. Absorbance was measured at 550 nm (test wavelength) and 655 nm (reference wavelength) using a microplate reader (Model 680; Bio-Rad Laboratories, Hercules, CA, USA). The results were expressed as the percentage of viability and calculated according to the following formula:


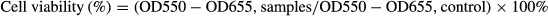


### *In vitro* functional differentiation assay

**Fig. 1:**
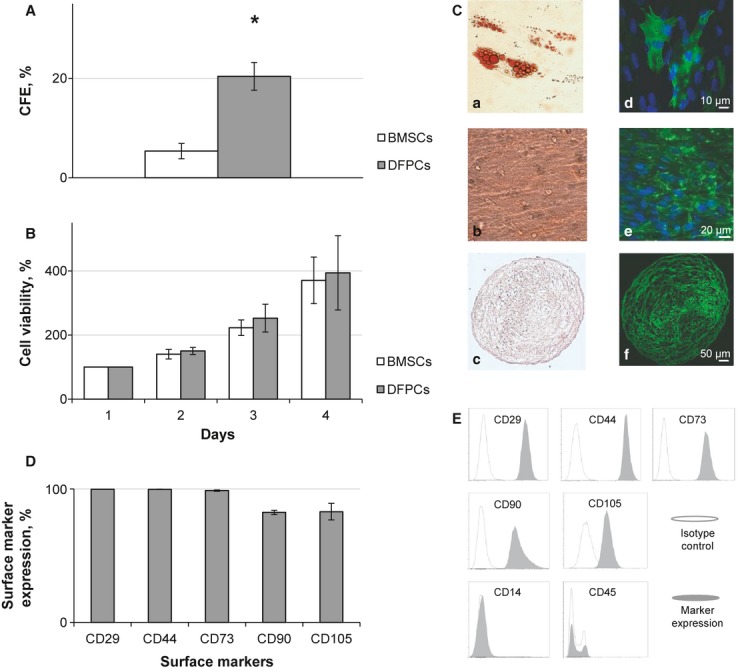
DFPCs possessed crucial stem cell properties. (**A**) DFPCs showed superior clonogenic capacity compared with BMSCs. Cell cultures were incubated for 12 days. Colonies containing more than 50 cells were scored as colonies. The percentage of colony-forming efficiency (CFE) was calculated according to the formula CFE (%) = (no. of colonies formed/no of cells seeded) ×100%; DFPCs *n* = 5, BMSCs *n* = 4, values represent the means ± SE, *P < 0.05 (Student's *t*-test). (**B**) Cell proliferation was similar for both cell types. Proliferation was assessed using the MTT dye reduction assay. Results were expressed as percentages of cell viability; DFPCs *n* = 5, BMSCs *n* = 4. (**C**) Multiple mesodermal lineage differentiation capacity of DFPCs *in vitro*. (a) Oil Red-O staining (Sigma-Aldrich, Buchs, Switzerland) of lipid vesicles performed 2 weeks after adipogenic stimulation, ×100. (b) Alizarin Red-S staining (Sigma-Aldrich) of hydroxyapatite-associated calcium mineral deposited in the extracellular matrix by osteoblastic cells derived by osteogenic differentiation, ×100. (c) Safranin O staining (Sigma-Aldrich) at day 21 after chondrogenic stimulation, indicating a homogeneous distribution of sulphated proteoglycans within the matrix structure, ×10. Adipogenesis (d), osteogenesis (e) and chondrogenesis (f) were additionally confirmed by immunostaining with fatty acid binding protein (FABP-4), osteocalcin and aggrecan, respectively (green). Nuclei were counterstained with DAPI (blue). (**D**) Surface marker expression in DFPCs was analysed by flow cytometry. DFPCs were positive for typical stem cell markers CD29, CD44, CD73, CD90 and CD105. No expression of haematopoietic markers CD14 and CD45 was detected. (**E**) Representative FACS histograms of CD29, CD44, CD73, CD90, CD105, CD14 and CD45 surface marker expression; *n* = 3 biological replicates**.**

The ability of human DFPCs to differentiate into multiple mesenchymal lineages was determined using a mesenchymal stem cell functional identification kit (R&D Systems, Minneapolis, MN, USA) according to the instructions of the manufacturer (Fig. 1).

### Fluorescence-activated cell sorter analysis

Dental follicle progenitor cells and BMSCs were analysed for epitope expression by fluorescence-activated cell sorter (FACS) analysis. Cells were incubated for 30 min at 4°C protected from light with saturating levels of the following monoclonal anti-human antibodies: CD14-V450, CD29-APC, CD44-PerCP-Cy5.5, CD45-V500, CD73-PE, CD90-biotin, V450-Streptavidin (BD Biosciences, Franklin Lakes, NJ, USA), CD105-Alexa Fluor 488 (AbD Serotec, Kidlington, UK), TLR2-FITC and TLR4-Alexa Fluor 488 (eBioscience, San Diego, CA, USA). FcR Blocking Reagent (human) (Miltenyi Biotec, Bergisch Gladbach, Germany) and buffer containing 0.5% bovine serum albumin were employed to reduce unspecific antibody binding. Isotype-matched antibodies served as controls. Cells were washed with PBS/EDTA (2 mM) and analysed using a LSR II Flow Cytometer (BD Biosciences). Dead cells were excluded using a dead cell staining kit (LIVE/DEAD®; Invitrogen, Carlsbad, CA, USA). Data analysis was performed with FACSDivaTM software (BD Biosciences). A minimum of 10,000 events were recorded per sample.

### LPS treatment and cytotoxicity assay

Ultrapure LPS from *P. gingivalis* was obtained from InvivoGen (San Diego, CA, USA) and used at final concentrations of 0, 1, 10 and 50 μg/ml in MSCGM. To determine the cytotoxic effects of LPS, MTT assays were performed as described above.

### RNA extraction and complementary DNA synthesis

Total RNA was extracted from cells using RNeasy Kit (Qiagen, Hilden, Germany). Genomic DNA contamination was eliminated by on-column digestion with RNase-free DNase (Qiagen). Complementary DNA was synthesized from 2 μg of total RNA using an oligo(dT)15 primer (Promega, Madison, WI, USA), 10 mM dNTPs (Invitrogen), rRNAsin ribonuclease inhibitor (Promega), and SuperScript® III Reverse Transcriptase (Invitrogen). Annealing was performed for 5 min at 65°C with rapid cooling at 4°C. Then reverse transcription was carried out for 60 min at 55°C, followed by 15 min at 70°C, with a final cool down to 4°C.

### Quantitative real-time PCR

**Fig. 2:**
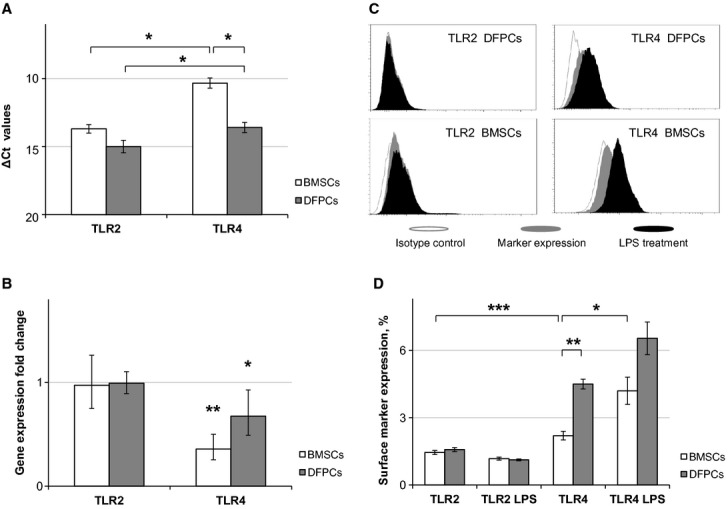
Effects of LPS on TLR2 and TLR4 expression in DFPCs and BMSCs. (**A**) Relative gene expression of TLR2 and TLR4 analysed by qRT-PCR. Relative gene expression of TLRs was determined based on the threshold cycle (C_T_) values. Only C_T_ values less than 35.5 were included. Results were normalized according to the formula: ΔC_T_ = C_T target gene_ − C_T GAPDH_. The scale is inverted, so that the higher histogram bars represent higher levels of mRNA; DFPCs *n* = 4, BMSCs *n* = 3, values represent the means ± SE, *P < 0.05 (Student's *t*-test). (**B**) Gene expression of TLR2 and TLR4 was down-regulated after LPS treatment in both DFPCs and BMSCs. ΔC_T_ values of samples were averaged and relative gene expression of LPS-treated cells(s) and calibrator(c) sample (*i.e*. untreated cells) were calculated following the delta/delta calculation method (2^−(ΔΔCt_s−ΔΔCt_c)^). Relative gene expression of the calibrator sample is always one. SE of normalized target gene expression relative to GAPDH was calculated from the initial SEs of the target gene and GAPDH. Each sample was tested in quadruplicate. Calculations were performed with Microsoft Excel®; DFPCs *n* = 4, BMSCs *n* = 3, values represent the means ± SE, *P < 0.05, **P < 0.01 (Student's *t*-test). (**C**) Protein expression of TLR2 and TLR4 in DFPCs and BMSCs was evaluated by flow cytometry. Representative FACS histograms of TLR2 and TLR4 expression are shown. (**D**) TLR2 and TLR4 were expressed at low levels on both DFPCs and BMSCs. The expression of TLR2 was significantly lower than TLR4. The TLRs expression level of DFPCs was not significantly influenced by LPS treatment, while the expression of TLR4 on LPS-treated BMSCs was elevated; DFPCs *n* = 5, BMSCs *n* = 4, values represent the means ± SE, *P < 0.05, **P = 0.01, ***P < 0.001 (Student's *t*-test).

Quantitative Real-Time PCR (qRT-PCR) was performed with StepOnePlus™ Real-Time PCR System (Applied Biosystems, Foster City, CA, USA) using TaqMan® Gene Expression Assays with TaqMan® Universal Master Mix, No AmpErase® UNG (Applied Biosystems) according to the instructions of the manufacturer. Reaction mixtures included specific primers for TLR2 (TaqMan® Gene Expression Assay ID: Hs01014511_m1; Applied Biosystems) and TLR4 (TaqMan® Gene Expression Assay ID: Hs00152939_m1; Applied Biosystems). Human GAPDH (TaqMan® Gene Expression Assay ID: Hs99999905_m1; Applied Biosystems) was used for normalization of each sample (housekeeping gene). Relative gene expression was calculated following the delta/delta calculation method (Fig. 2B).

### *In vitro* wound healing assay

For wounding, DFPCs and BMSCs were cultured in 24-well plates until they reached 90% confluence. Afterwards, a disposable plastic (200 μl) pipette tip was used to prepare a scratch across the monolayer of cells. Intact cells were gently washed twice with PBS to remove debris created by ‘wounding’ and culture medium was added for the remainder of incubation. The extent of repopulation of the wound area was assessed for up to 24 hrs by live imaging, processed by ELYRA PS.1 LSM-780 (Carl Zeiss). Images were captured every 3 min, thus allowing the observation of the healing process *in vitro*, in which the cells at the edges of the artificial wound migrated towards the wound area. The average wound dimensions were measured using AxioVision Rel 4.5 SP1 software (Carl Zeiss). Rates of healing were calculated at several time points and normalized to untreated controls.

### Detection of IL-6 by enzyme linked immunosorbent assay (ELISA)

Supernatants were collected from LPS-treated as well as untreated DFPCs and BMSCs and analysed for IL-6 secretion by a commercially available sandwich ELISA kit (ImmunoTools, Friesoythe, Germany) according to the instructions of the manufacturer. Serial dilutions of human recombinant IL-6 standard were included in each assay to obtain a standard curve. Absorbance was measured at a wavelength of 450 nm using a microplate reader (Bio-Rad Laboratories).

### Statistical analysis

All results are presented as means ± standard error (SE). Statistical analyses were carried out by *t*-test (SigmaStat 3.5; Systat Software Inc., San Jose, CA, USA). Differences were considered statistically significant at *P* < 0.05.

## Results

### DFPCs possessed crucial stem cell properties

In this study, we isolated human DFPCs from freshly extracted dental follicle tissues by their ability to adhere to a plastic substratum. Adherent fibroblast-like cells grew in a stem cell growth medium and began to form colonies. Human BMSCs were isolated from aspirates of bone marrow, also by plastic adherence and cultured under the same conditions as DFPCs. Evaluation of CFE was possible for both cell populations after the first passage. The CFE of cells derived from dental follicle tissue (20.4 ± 2.8%) was significantly higher (*P* < 0.05) compared to BMSCs CFE (5.3 ± 1.5%) (Fig. 1A). DFPCs metabolic activity and proliferation did not differ significantly from those of BMSCs (Fig. 1B). Furthermore, DFPCs had the capacity to differentiate into different mesodermal lineages (Fig. 1C) and exhibited a strong positive expression of several surface markers typical for stem cells (CD29, CD44, CD73, CD90 and CD105) (Fig. 1D and E).

### DFPCs were able to sense *P. gingivalis* LPS

To investigate the expression of TLR2 and TLR4 in DFPCs and BMSCs, qRT-PCR and flow cytometry were used. Data analysis revealed the presence of TLR2 and TLR4 mRNA in both cell populations. Specifically, TLR4 expression was significantly higher than TLR2 expression (*P* < 0.05). Moreover, TLR4 gene expression was higher in BMSCs compared to DFPCs (*P* < 0.05) (Fig. 2A). We also tested TLR2 and TLR4 gene expression in cells stimulated for 24 hrs by 50 μg/ml *P. gingivalis* LPS. This high dose LPS treatment did not influence the expression level of TLR2 mRNA, whereas the gene expression of TLR4 was significantly down-regulated (*P* < 0.05) (Fig. 2B). In addition, we confirmed that DFPCs and BMSCs expressed TLR2 and TLR4 at protein level. Concretely, both cell populations expressed low levels of TLR2 and TLR4 (Fig. 2C). The expression of TLR2 on DFPCs was significantly lower than TLR4 expression (*P* < 0.001). The expression of TLR4, but not TLR2, was elevated on LPS-treated BMSCs (*P* < 0.05). Interestingly, TLR2 and TLR4 expression on DFPCs was not significantly affected by LPS treatment (Fig. 2D).

### LPS promoted migration of DFPCs

**Fig. 3:**
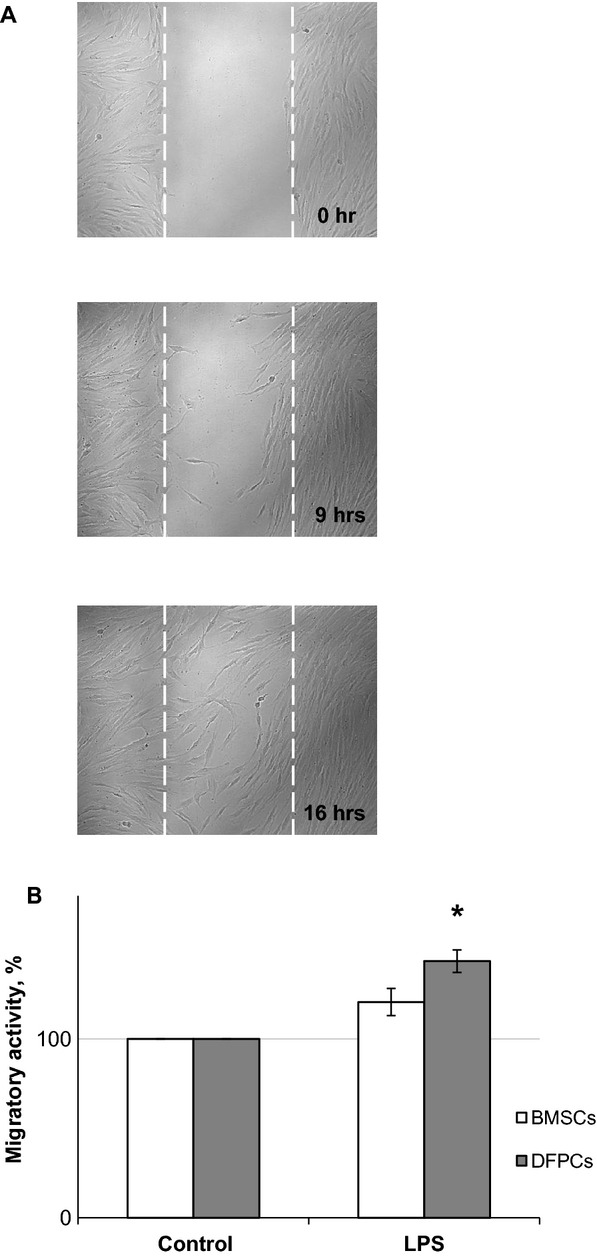
Effects of LPS on the migration rates of DFPCs and BMSCs. (**A**) *In vitro* wound healing assay. After scratching a confluent cell monolayer, surrounding cells migrated into the wound area (time after scratching is indicated). Dotted lines in images represent wound edges at *t* = 0 hr, ×10. (**B**) LPS treatment significantly increased the migratory activity of DFPCs. Rates of healing were calculated from average wound dimensions at several time points and normalized to untreated controls. Results are presented as mean percentages; DFPCs *n* = 4, BMSCs *n* = 3, values represent the means ± SE, *P < 0.05 (Student's *t*-test).

To test whether LPS affects cell migration, confluent DFPC as well as BMSC cultures were subjected to an *in vitro* wound healing assay. Cells were either left untreated or stimulated for 72 hrs with 50 μg/ml *P. gingivalis* LPS. Data analysis indicated that cells migrated in a linear fashion. As shown in Figure 3, LPS-treated DFPCs had a 43.5% higher migratory capacity compared to untreated controls (*P* < 0.05), suggesting that LPS promotes DFPCs basal motility. BMSCs migration was also enhanced (20.7%), although not significantly (*P* > 0.05).

### LPS had no cytotoxic effects on DFPCs

**Fig. 4:**
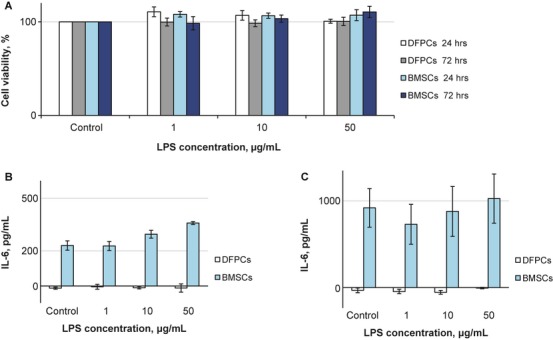
Effects of LPS on cell viability and IL-6 secretion. (**A**) LPS treatment showed no cytotoxic effects on either DFPCs or BMSCs. Series of MTT assays were performed to determine cell viability. Cells were treated with ultrapure *Porphyromonas gingivalis* LPS for 24 and 72 hrs; DFPCs *n* = 7, BMSCs *n* = 6. Histograms show IL-6 secretion by DFPCs and BMSCs, measured after 24 hrs (**B**) and 72 hrs (**C**) of LPS stimulation by a commercially available sandwich ELISA kit. Limit of detection was 8 pg/ml. Each sample was tested in triplicate; DFPCs *n* = 3, BMSCs *n* = 5, values represent the means ± SE, *P* > 0.05 (Student's *t*-test).

To verify whether LPS evokes cytotoxicity effects on DFPCs and BMSCs, we examined cell viability by MTT assay. Interestingly, the cell viability of both populations was not influenced, even when cells were treated with a high LPS dosage (50 μg/ml) or for a long time period (72 hrs) (Fig. 4A).

### LPS did not influence the secretion of IL-6 by DFPCs

Next, we examined whether LPS induced IL-6 secretion by examining culture supernatants of both DFPCs and BMSCs. No IL-6 could be measured in any of the DFPC culture supernatants. The detected signal did not exceed that of medium control in any of the tested samples. On the contrary, BMSCs produced IL-6. Cytokine secretion was detected after 24 and 72 hrs of treatment and was independent of the LPS dosage (*P* > 0.05) (Fig. 4B and C).

## Discussion

In this study, human DFPCs and BMSCs were isolated from dental follicle tissues of wisdom teeth and bone marrow, respectively, by applying methodology that had been previously developed 8, 16. As the impact of bacteria and bacterial components on stem cell functions is of high interest for periodontal regenerative medicine, we investigated the influence of LPS on gene expression, migratory ability, cell viability and cytokine production of DFPCs and BMSCs.

In an effort to understand cells responsiveness to LPS, we have analysed the TLR2 and TLR4 mRNA expression. Recent reports indicated that BMSCs express TLR proteins, which are believed to play a critical role in immunomodulation 17, 18, 19, 20. Tomic *et al*. reported gene expression of TLR3 and TLR4 on human DFPCs 10. Here we report for the first time the expression of TLR2 and TLR4 on human DFPCs at both mRNA and protein level. Notably, TLR2 gene expression was not affected by the LPS treatment, whereas the expression of TLR4 was significantly down-regulated in both DFPCs and BMSCs. Interestingly, the protein expression of both TLRs on DFPCs remained at low levels even under the influence of LPS. These results show that DFPCs are able to express TLR4, a receptor reported to be responsible for LPS sensing 14. We suggest that TLR4 mRNA down-regulation may be part of an adaptive mechanism of cells being exposed to bacteria, as already proposed 9. We also support the notion that TLR2 recognizes mainly lipoproteins and lipopeptides rather than LPS 21.

The development of effective therapies for periodontitis involves the engraftment of multipotent cells in sites of periodontal tissue destruction 22. The tissue regeneration potential appears to be dependent on the management of repopulation and healing of periodontal defects 23. Thus, factors favouring cell migration have been in focus of current research. Several studies have already described an enhancement of MSCs mobility after stimulation with TLR agonists. Waterman *et al*. suggest that MSC polarization could explain the effect of TLR stimulation and its downstream consequences on the migratory properties of stem cells 24. Another study on human BMSCs showed that stimulation of BMSCs with TLR agonists led to the activation of downstream signalling pathways, including NF-κB, AKT and MAPK. 25. Park *et al*. demonstrated that LPS promoted the migration of murine odontoblast-like cells *via* TLR4 through the ERK and PI3/AKT signalling pathways 26. Here, we sought to investigate the effect of TLR stimulation on the migratory ability of DFPCs and BMSCs using an *in vitro* wound healing model. Interestingly, LPS-treated treatment DFPCs showed a significantly higher migratory activity than the untreated controls, whereas the influence of LPS on the migration rates of BMSCs was not statistically significant. These data suggest a positive impact of LPS on the mobility of DFPCs, which could play a pivotal role in tissue repair processes.

Reports in the literature suggest that oral bacterial biofilms may contain more than 10^5^ microorganisms 27, while the concentrations and compositions of pathogenic bacteria in the subgingival biofilm vary greatly depending on the local micro-environmental conditions 28. Based on these reports, we decided to use high *P. gingivalis* LPS concentrations, which could resemble the LPS concentrations likely to be found in the subgingival plaque of periodontal pockets. According to our results, cell viability of both DFPCs and BMSCs was not affected by LPS treatment. This could be explained by the low endotoxic potency of *P. gingivalis* LPS in comparison to lipopolysaccharides derived from enteric bacteria, like *Escherichia coli* 29, 30. Specifically, it has been demonstrated that *P. gingivalis* LPS possesses a lipid A with a markedly distinct structure, thus differing from enterobacterial LPS in its ability to elicit a variety of responses 31, 32.

Furthermore, we investigated the IL-6 secretion by DFPCs and BMSCs. IL-6 is a multifunctional cytokine, which is involved in the regulation of the host immune response to periodontal pathogens, leading to local and systemic inflammatory reactions 33. Moreover, recent studies demonstrated that IL-6 could act in a paracrine fashion enhancing the migratory potential of MSCs 34, 35. Signalling pathways that control migration of MSCs, involve various important molecular mechanisms, including chemoattractant-receptor axes and intracellular signalling cascades 36. In this study, we focused on the possible role of IL-6 in the migration of DFPCs. Notably, the analysis of IL-6 secretion showed no cytokine production by DFPCs. This could imply that DFPCs may not actively participate in the initiation of inflammatory processes and retain their neutral character even under the influence of toxins. Furthermore, it seems reasonable to suppose that the enhanced migratory activity of DFPCs is regulated independently of IL-6. On the contrary, we have proven the secretion of IL-6 by BMSCs. Nevertheless, the cytokine production was not significantly affected by the LPS treatment. We suggest that *P. gingivalis* LPS may not significantly influence the intracellular signalling cascades that give rise to IL-6 production by either DFPCs or BMSCs.

In sum, the above data indicate that DFPCs represent a progenitor cell population with unique properties but also similarities with other multipotent cells, such as BMSCs. DFPCs showed a stem cell phenotype similar to BMSCs but possessed higher clonogenic capacity. We demonstrated that DFPCs, as BMSCs, expressed both TLR2 and TLR4. Notably, the migratory potential of DFPCs was significantly elevated after high dosage LPS treatment and was not IL-6 driven. On the contrary, LPS stimulation did not induce pro-inflammatory cytokine secretion by DFPCs. Thus, according to our results we hypothesize that even though DFPCs can sense bacterial components, they may not play an active role in the initiation of the immune response of the host. On the other hand, their high clonogenic efficiency and their enhanced migratory capacity under the influence of toxins could signal their superior regeneration potential and distinguish their fate from that of other populations of multipotent cells. Future studies on the migration mechanisms and the multilineage differentiation capacity of DFPCs in the presence of LPS or other bacterial components are necessary to support this tempting theory. In conclusion, the above findings support the potential of using human DFPCs as biological graft for periodontal regenerative therapies.

## References

[1] Van Dyke TE (2008). The management of inflammation in periodontal disease. J Periodontol.

[2] Silvério KG, Benatti BB, Casati MZ (2008). Stem cells: potential therapeutics for periodontal regeneration. Stem Cell Rev.

[3] Wang HL, Greenwell H, Fiorellini J (2005). Periodontal regeneration. J Periodontol.

[4] Honda MJ, Imaizumi M, Tsuchiya S (2010). Dental follicle stem cells and tissue engineering. J Oral Sci.

[5] Lin NH, Gronthos S, Bartold PM (2008). Stem cells and periodontal regeneration. Aust Dent J.

[6] Huang GT, Gronthos S, Shi S (2009). Mesenchymal stem cells derived from dental tissues *vs*. those from other sources: their biology and role in regenerative medicine. J Dent Res.

[7] Ten Cate AR (1997). The development of the periodontium-a largely ectomesenchymally derived unit. Periodontol 2000.

[8] Morsczeck C, Götz W, Schierholz J (2005). Isolation of precursor cells (PCs) from human dental follicle of wisdom teeth. Matrix Biol.

[9] Mo IF, Yip KH, Chan WK (2008). Prolonged exposure to bacterial toxins downregulated expression of toll-like receptors in mesenchymal stromal cell-derived osteoprogenitors. BMC Cell Biol.

[10] Tomic S, Djokic J, Vasilijic S (2011). Immunomodulatory properties of mesenchymal stem cells derived from dental pulp and dental follicle are susceptible to activation by toll-like receptor agonists. Stem Cells Dev.

[11] Botero TM, Son JS, Vodopyanov D (2010). MAPK signaling is required for LPS-induced VEGF in pulp stem cells. J Dent Res.

[12] Bostanci N, Belibasakis GN (2012). *Porphyromonas gingivalis*: an invasive and evasive opportunistic oral pathogen. FEMS Microbiol Lett.

[13] Kocgozlu L, Elkaim R, Tenenbaum H (2009). Variable cell responses to *P. gingivalis* lipopolysaccharide. J Dent Res.

[14] Darveau RP, Pham TT, Lemley K (2004). *Porphyromonas gingivalis* lipopolysaccharide contains multiple lipid A species that functionally interact with both toll-like receptors 2 and 4. Infect Immun.

[15] DelaRosa O, Lombardo E (2010). Modulation of adult mesenchymal stem cells activity by toll-like receptors: implications on therapeutic potential. Mediators Inflamm.

[16] Gaebel R, Furlani D, Sorg H (2011). Cell origin of human mesenchymal stem cells determines a different healing performance in cardiac regeneration. PLoS ONE.

[17] Hwa Cho H, Bae YC, Jung JS (2006). Role of toll-like receptors on human adipose-derived stromal cells. Stem Cells.

[18] Pevsner-Fischer M, Morad V, Cohen-Sfady M (2007). Toll-like receptors and their ligands control mesenchymal stem cell functions. Blood.

[19] Liotta F, Angeli R, Cosmi L (2008). Toll-like receptors 3 and 4 are expressed by human bone marrow-derived mesenchymal stem cells and can inhibit their T-cell modulatory activity by impairing Notch signaling. Stem Cells.

[20] Raicevic G, Rouas R, Najar M (2010). Inflammation modifies the pattern and the function of toll-like receptors expressed by human mesenchymal stromal cells. Hum Immunol.

[21] Jin MS, Lee JO (2008). Structures of the toll-like receptor family and its ligand complexes. Immunity.

[22] Ivanovski S, Gronthos S, Shi S (2006). Stem cells in the periodontal ligament. Oral Dis.

[23] Polimeni G, Xiropaidis AV, Wikesjö UM (2006). Biology and principles of periodontal wound healing/regeneration. Periodontol 2000.

[24] Waterman RS, Tomchuck SL, Henkle SL (2010). A new mesenchymal stem cell (MSC) paradigm: polarization into a pro-inflammatory MSC1 or an Immunosuppressive MSC2 phenotype. PLoS ONE.

[25] Tomchuck SL, Zwezdaryk KJ, Coffelt SB (2008). Toll-like receptors on human mesenchymal stem cells drive their migration and immunomodulating responses. Stem Cells.

[26] Park JH, Kwon SM, Yoon HE (2011). Lipopolysaccharide promotes adhesion and migration of murine dental papilla-derived MDPC-23 cells *via* TLR4. Int J Mol Med.

[27] Jain S, Darveau RP (2010). Contribution of *Porphyromonas gingivalis* lipopolysaccharide to periodontitis. Periodontol 2000.

[28] Socransky SS, Haffajee AD (2005). Periodontal microbial ecology. Periodontol 2000.

[29] Dixon DR, Darveau RP (2005). Lipopolysaccharide heterogeneity: innate host responses to bacterial modification of lipid a structure. J Dent Res.

[30] Muthukuru M, Jotwani R, Cutler CW (2005). Oral mucosal endotoxin tolerance induction in chronic periodontitis. Infect Immun.

[31] Millar SJ, Goldstein EG, Levine MJ (1986). Modulation of bone metabolism by two chemically distinct lipopolysaccharide fractions from *Bacteroides gingivalis*. Infect Immun.

[32] Ogawa T, Asai Y, Makimura Y (2007). Chemical structure and immunobiological activity of *Porphyromonas gingivalis* lipid A. Front Biosci.

[33] Nibali L, Fedele S, D'Aiuto F (2012). Interleukin-6 in oral diseases: a review. Oral Dis.

[34] Rattigan Y, Hsu JM, Mishra PJ (2010). Interleukin 6 mediated recruitment of mesenchymal stem cells to the hypoxic tumor milieu. Exp Cell Res.

[35] Schmidt A, Ladage D, Schinköthe T (2006). Basic fibroblast growth factor controls migration in human mesenchymal stem cells. Stem Cells.

[36] Li L, Jiang J (2011). Regulatory factors of mesenchymal stem cell migration into injured tissues and their signal transduction mechanisms. Front Med.

